# Prevalence of canine leishmaniasis in Beichuan County, Sichuan, China and phylogenetic evidence for an undescribed *Leishmania* sp. in China based on 7SL RNA

**DOI:** 10.1186/1756-3305-5-75

**Published:** 2012-07-12

**Authors:** Ke Sun, Wang Guan, Jian-Guo Zhang, Ya-Jing Wang, Yu Tian, Lin Liao, Bin-Bin Yang, Da-Li Chen, Jian-Ping Chen

**Affiliations:** 1 Department of Parasitology, School of Preclinical and Forensic Medicine, Sichuan University, Chengdu, Sichuan, 610041, China; 2 Department of Parasitology, North Sichuan Medical College, Nanchong, Sichuan, 637000, China; 3 Animal Disease Prevention and Food Safety Key Laboratory of Sichuan Province, Sichuan University, Chengdu, Sichuan, 610064, China; 4 Institute of Laboratory Medicine, Ningxia Medical University, Yinchuan, 750004, China

**Keywords:** Prevalence, Canine leishmaniasis, Phylogeny, rK39 dip-stick, Enzyme-linked immunosorbent assay (ELISA), 7SL RNA

## Abstract

**Background:**

Leishmaniasis is a vector-borne disease, which is still endemic in the west and northwest area of China. Canines are the major reservoirs of *Leishmania,* the etiological agent of human visceral leishmaniasis. *Phlebotomus chinensis* is the main transmission vector of zoonotic visceral leishmaniasis (ZVL).

**Methods:**

In this study, rK39 dip-stick, ELISA and PCR methods were used to investigate the prevalence of canine leishmaniasis (CanL) in Beichuan County, Sichuan Province, China.

**Results:**

Among the 86 dogs which were included in the study, 13 dogs were positive using the dip-stick test (15.12%), while 8 dogs were positive using ELISA (9.30%) and 19 dogs were positive for PCR (22.03%). In total, 32 dogs were positive for one or more tests (37.21%). Interestingly, phylogenetic analysis based on the partial 7SL RNA fragment provided evidence that an undescribed *Leishmania* species, which is clearly a causative agent of CanL and human visceral leishmaniasis, does exist in China. This result is consistent with our previous study.

**Conclusions:**

Our work confirmed that canine leishmaniasis is still prevalent in Beichuan County. Further control is urgently needed, as canine leishmaniasis is of great public health importance. The phylogenetic analysis based on 7SL RNA segment provides evidence for the existence of an undescribed *Leishmania* sp. in China.

## Background

Leishmaniasis is a vector-borne disease caused by different species of the genus *Leishmania,* including subgenera *Viannia* and *Leishmania.* The diseases are characterized by a spectrum of clinical manifestations: cutaneous leishmaniasis (CL), mucocutaneous leishmaniasis (MCL) and visceral leishmaniasis (VL) [1]. Globally, leishmaniasis affects 88 countries, which is an estimated 500,000 cases of VL and 1–1.5 million cases of CL each year [2]. Epidemiologically, canines are the major reservoirs of *Leishmania*, the etiological agent of human visceral leishmaniasis [3-7]. Following the accelerating urbanization and population mobility, canine leishmaniasis is epidemic in America, Asia and Europe [8-14].

Leishmaniasis is still endemic in China, especially in the west and northwest regions. The prevalence of human leishmaniasis in west China was alarming according to the reports of Wang *et al*. [15] and Qu *et al*. [16]. Moreover, as our previous study [17,18] indicated, *Leishmania* species that are endemic in China is complicated since several species have been reported, including *L. donovaniL. infantumL. gerbilli* and *L. turanica*. Interestingly, an undescribed *Leishmania* species may exist in China, which was reported by Cao *et al*. [17], Yang *et al*. [18] and Guan *et al*. [19] using molecular methods. The Dog is the major reservoir in Sichuan province, which belongs to the zoonotic visceral leishmaniasis (ZVL) area [20]. Several sand fly species have been shown to be related to zoonotic visceral leishmaniasis ZVL in the epidemic area of Sichuan Province, such as *Phlebotomus chinensisSergentomyia koloshanensisSergentomyia barraudi* and *Sergentomyia squamirostris*[21]. The number of stray dogs increased after the earthquake that occurred in Wenchuan County, China, on May 12th, 2008, which increased the risk of leishmaniasis outbreaks. Beichuan County was one of the worst affected counties (in terms of destruction) in the Wenchuan great earthquake and the epidemiology of canine leishmaniasis (CanL) has not been reported. Therefore, it is a matter of urgency to survey the prevalence of canine leishmaniasis in Beichuan County, Sichuan, China [22].

Most infected dogs do not show clinical signs. These asymptomatic dogs harbor the parasite, and thus play an important role in the maintenance of the etiological agent of human visceral leishmaniasis [23,24]. Serological analyses and PCR methods have been successfully used to detect *Leishmania* infection in symptomatic and asymptomatic dogs [8-11,25]. Although sero-positivity is found to be very high in symptomatic dogs, it is inefficient in diagnosis of the asymptomatic ones [26,27]. PCR assay can greatly enhance the sensitivity of the diagnosis of *Leishmania* infection in asymptomatic dogs [25]. The 7 spliced leader (7SL) RNA is a component of the signal recognition particle in eukaryotes [28]. It has been reported that 7SL RNA is highly conserved and can be used to differentiate *Leishmania* species [29-31].

Thus, in this study we utilized rK39 dip-stick, ELISA and PCR methods targeting 7SL RNA gene to investigate the prevalence of canine leishmaniasis in Beichuan County, Sichuan Province, China. For accurate identification of the *Leishmania* species prevalent in this area, we further performed phylogenetic inference on the basis of 7SL RNA segments analysis.

## Methods

### Study area

**Figure 1:**
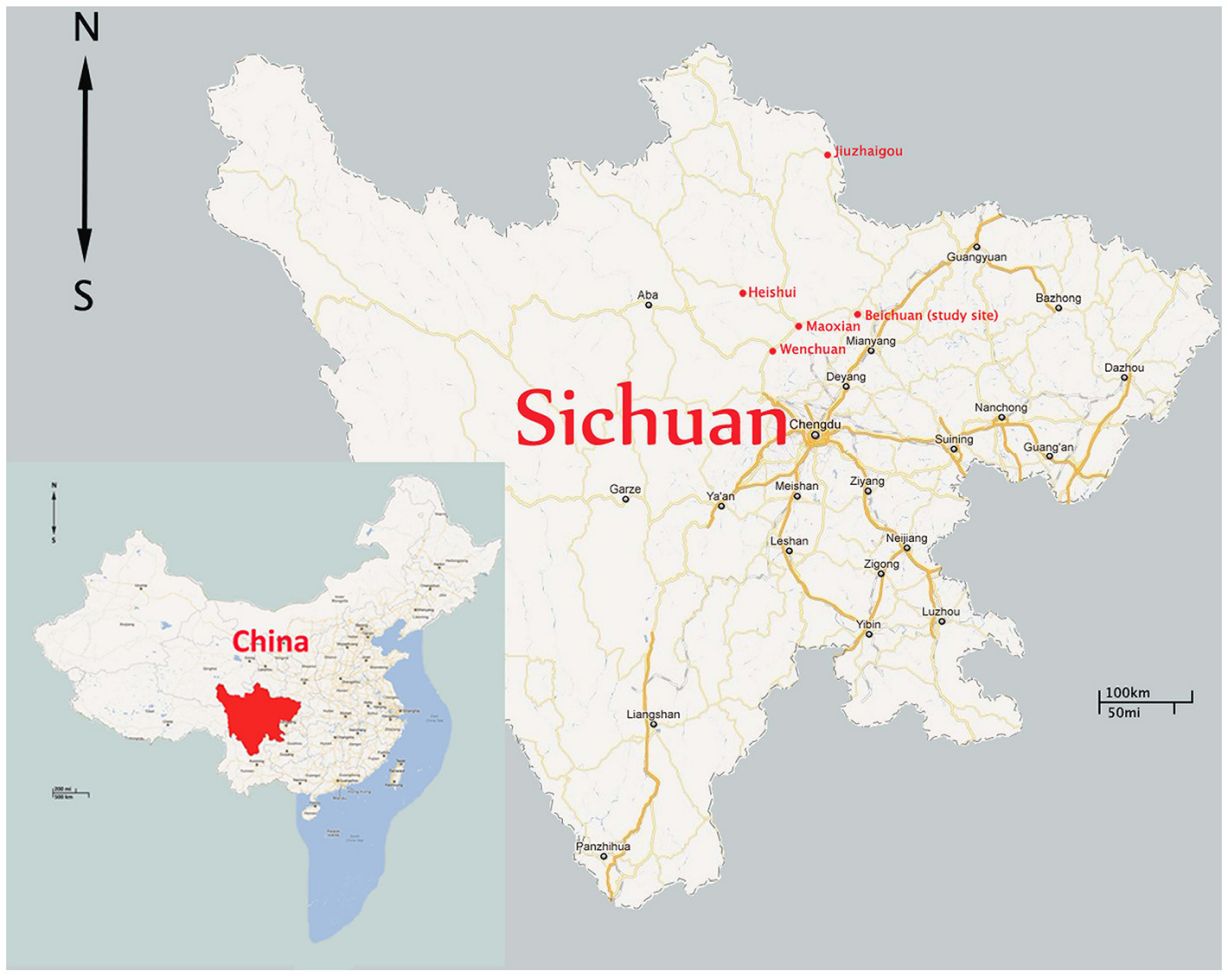
**The map of China showing the study area located in the northwest of Sichuan province.** The red color marked places are canine leishmaniasis endemic areas.

Beichuan County (31° 14′ ~ 32° 14′N, 103° 44′ ~ 104° 42′E) is located in the northwest of Sichuan province (Figure 1). Three villages, Mazao, Badi and Dunshang, were chosen to carry out this study. These villages are located in a mountainous area, 800–1200 m above sea level. Houses were rebuilt after Wenchuan great earthquake along the mountains. The population is composed mainly of peasants. Almost every family raised at least one dog as house guardian. Dogs were not allowed to enter the house, and some of them were tied nearby the house, other dogs became stray dogs due to the Wenchuan great earthquake. This county is a known leishmaniasis endemic area, with 1–2 human VL cases reported every year.

### Animals and sampling procedures

Samples were collected from dogs during field trips in September 2011. The study on dogs was approved by the Ethical Review Committee of the National Institute of Parasitic Disease, Chinese Center for Disease Control and Prevention (CDC) in Shanghai. Dogs were sampled in 81 houses where oral informed consent was granted. A total of 86 dogs, numbered as Canine-1 to Canine-86 by the phlebotomized succession, were concluded and their age, gender, clinical signs compatible with CanLwere registered [3]. Among the 86 dogs, 7 dogs were from Macao while 64 dogs were from Badi which was the most severe CanL endemic village and 15 dogs were from Dunshang. 2 ml blood samples were obtained from each dog by venipuncture of the foreleg vein, and then numbered according to the dog number. 1 ml blood was stored in sterile, EDTA-coated tubes to extract parasite DNA for PCR tests. The other 1 ml blood was placed in sterile tubes, which were free of anticoagulant, then centrifuged at 2800 rpm for 20 minutes. Sera were collected to detect the specific antigen of *Leishmania*. All samples were then stored at −20°C.

### Serological analysis

#### rK39 dip-stick test

All 86 samples were tested for anti-rK39IgG antibodies in the field using the Kalazar Detect rK39 dip-stick (InBios, USA) according to the manufacturer’s protocol.

#### ELISA test

A double antibody sandwich method was performed using the Canine leishmaniasis kit (R&D, USA) to detect the *Leishmania* antigen in the serum according to the manufacturer’s protocol.

### Molecular techniques

#### DNA extraction

DNA was extracted from dog blood samples using Tiangen Blood DNA Kit (Tiangen, China) according to manufacturer’s protocol. *Leishmania* strain MHOM/CN/90/SC10H2 was chosen as the positive control and has previously been shown to induce extracellular amastigote transformation by Cao *et al*. [32]. The parasite was cultured in NNN medium at 24°C for 7 days, then amplified in culture in medium 199 supplemented with 15% heat-inactivated fetal bovine serum at 25°C. Approximately 1 × 10^9^–5 × 10^9^ promastigotes were collected by centrifugation at 4,000 rpm for 10 min at 4°C and washed with PBS. Total genomic DNA was extracted from the promastigotes using a standard sodium dodecyl sulfate–proteinase K procedure described by Sambrook and Russell [33].

#### Detection of genomic DNA by PCR

The 7SL gene was amplified using the primers described by Zelazny *et al*. [31]. Primer TRY7SL.For1.M13 (5′-GTA AAA CGA CGG CCA GTG CTC TGT AAC CTT CGG GGG CT-3′) and TRY7SL. Rev1. M13 (5′-CAG GAA ACA GCT ATG ACG GCT GCT CCG TYN CCG GCC TGA CCC-3′) were commercially synthesized and dissolved in nuclease free water to give a working concentration of 10 pMol. The PCR tests were performed in Peltier Thermal Cycler (BIO-RAD, USA) with a reaction mixture consisting of 0.5μL Golden Fast DNA Polymerase (Tiangen, China), 12μL 2 × Golden Fast Reaction Mix,1μL of forward primer, 1μL of reverse primer, 3μL of extracted DNA, and ultra-pure water to give a final volume of 25μL. The PCR thermo cycling program consisted of an initial step at 95°C for 5 minutes, followed by 36 cycles at 95°C for 15 seconds, 65°C for 5 seconds, and 72°C for 15 seconds, and a final incubation at 72°C for 2 minutes. The PCR products were analysed on a 1.5% agarose gel stained with ethidium bromide then using a DNA purification kit (Omega, USA) following the manufacturer’s protocol. Purified products were sequenced using an ABI BigDye Terminator chemistry on an ABI 3730 automated sequencer with the primer M13 Forward (5′-GTAAAACGACGGCCAG-3′) and M13 Reverse (5′-CAGGAAACAGCTATGAC-3′). The determined sequences were then submitted to GenBank.

#### Phylogenetic analyses

A set of 7SL RNA sequences of *Leishmania* were retrieved from GenBank, including twenty two strains of subgenus *Leishmania*, eight strains of subgenus *Viannia*, one strain of *Leishmania tarentolae*. *Trypanosoma brucei* was included as an outgroup. The sequences were aligned using Clustal X 1.83 [34] with default gap penalties. The p-distance matrices were computed with MEGA v.4.0.2 [35].

Phylogenetic hypotheses of *Leishmania* were generated with 7SL RNA segments using Bayesian inference (BI) with the program MrBayes v.3.2 [36]. Gaps were treated as missing data. *T. brucei* was used to root the trees according to a recent study on the phylogeny of Trypanosomatidae [37]. Prior to phylogenetic analyses, the best-fit model of evolution, GTR + G, was selected using jModelTest v.0.1.1 [38] under the Akaike information criterion [39] following recent recommendations [40]. Posterior probability distribution was estimated by allowing four incrementally heated Markov chains to proceed for four million times, with samples taken from every 200 generations. To ensure our analyses were not restricted from the global optimum, analyses were repeated beginning with different starting trees [41]. The first one million generations were discarded in case this chain reached stationary, and the remaining samples from the independent runs were pooled to obtain the final approximation of the posterior distribution of trees. The posterior distribution was summarized as a 50% majority-rule consensus to form a robust phylogeny. The results of Bayesian analyses were accessed with Treeview v1.6.6 [42].

## Results

### Serological and PCR detection results

A total of 86 dogs were tested. All tested dogs were older than 4-months (going through at least one sand fly season during May to September), males accounted for 64/86 (74.42%). None of them presented any clinical signs of CanL. Results of serological tests (including dipstick test and ELISA), PCR are shown in Table 1. 13 dogs were positive for Dipstick test (15.12%), while 8 dogs were positive for ELISA test (9.30%) and 19 dogs were positive for PCR (22.03%). The prevalence in Macao, Badi, Dunshang were 1/7(14.29%), 27/64(42.19%), 4/15(26.67%) respectively. Globally, 32/86 (37.21%) dogs were positive for one or more tests.

**Table 1:** Serology and PCR detection results in dogs living in the three villages, Mazao, Badi and Dunshang

Dipstick	ELISA	PCR	No of dogs in Maozao	No of dogs in Badi	No of dogs in Dunshang	Total
+	+	+	0	1	0	1
+	+	-	0	0	0	0
+	-	-	1	3	3	7
-	+	+	0	1	0	1
-	+	-	0	6	0	6
+	-	+	0	5	0	5
-	-	+	0	11	1	12
-	-	-	6	37	11	54
Total			7	64	15	86

### DNA sequences and phylogenetic analyses

All the PCR-positive samples were sequenced, and numbered as canine-10 to canine-72 according to the dog number. 7SL RNA of the strain MHOM/CN/90/SC10H2, which was isolated from human patients in 1990 in Sichuan province was also obtained. GenBank accession numbers of these sequences were **JQ315203** to **JQ315222**. After alignment by Clustal X 1.83, some minor adjustment of the aligned matrix was done by Mega 4.0. Prior to the Bayesian analysis, the most adequate model of evolution GTR + G (−ln *L* = 530.5067) was selected by jModeltest.

**Figure 2:**
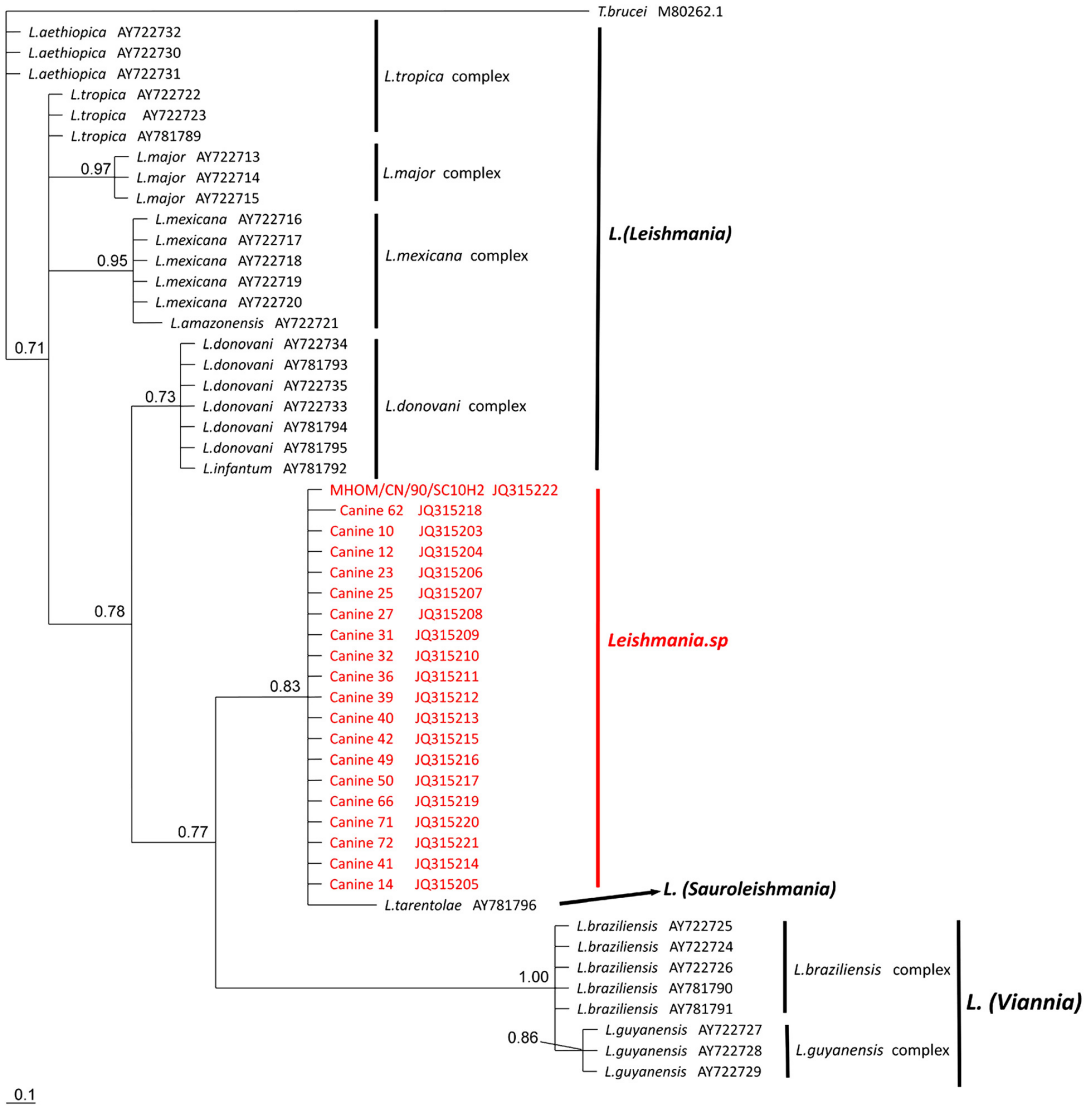
**The 50% majority-rule consensus tree inferred from Bayesian inference of 7sl dataset using MrBayes v. 3.2.** Numbers at nodes represent Bayesian posterior probabilities.

For the aligned matrix of 7SL RNA, overall mean p-distance was 0.096. The p-distance between each complex was also computed (Table 2). In the phylogenetic tree inferred from this matrix (Figure 2), a clear division was observed between the two subgenera *Leishmania* and *Viannia*. Surprisingly, the unisolated *Leishmania* in our study and strain MHOM/CN/90/SC10H2 formed a clade with nonpathogenic *L. tarentolae* (PP = 0.83) rather than any other pathogenic *Leishmania* species, thus termed *Leishmania sp*. The p-distance within this group was 0.00924. To the other complex, the p-distance ranged from 0.02935 (*L. tarentolae*) to 0.18022 (*L. guyanensis* complex).

**Table 2:** **Pairwise genetic distances for 7SL RNA segments among*Leishmania*strains in this study**

		1	2	3	4	5	6	7	8
1	*Leishmania sp.*								
2	*T. brucei*	0.30043							
3	*L. tropica* complex	0.09526	0.25758						
4	*L. tarentolae*	0.02935	0.27068	0.12409					
5	*L. mexicana* complex	0.08086	0.25824	0.05184	0.09512				
6	*L. major* complex	0.10255	0.26515	0.03650	0.13139	0.07441			
7	*L. donovani* complex	0.06565	0.27850	0.05846	0.09430	0.06591	0.06576		
8	*L. guyanensis* complex	0.18022	0.32090	0.18978	0.20290	0.18046	0.18248	0.17305	
9	*L. braziliensis* complex	0.17302	0.31343	0.18248	0.19565	0.17438	0.17518	0.16684	0.00719

## Discussion

Leishmaniasis is still endemic in Sichuan, China, with over 100 cases reported every year [32]. The environment was vastly altered due to the Wenchuan great earthquake in 2008. As a result of that, stray dogs were increasing. Similar studies focusing on Jiuzhaigou County, Wenchuan County and Heishui County of Sichuan Province indicated that, the rate of positive dogs for CanL tests was high, especially in asymptomatic dogs, which highlights the risk of the potential leishmaniasis outbreaks in these counties [20,43]. As Beichuan County is geographically adjacent to Jiuzhaigou County, Wenchuan County, it is also very important to investigate the prevalence of CanL in this county.

In this study, a total of 86 dogs were tested for CanL using rK39 dip-stick, ELISA and PCR methods. PCR targeting 7SL RNA presented a higher positive rate than rK39 dip-stick and ELISA methods (22.03% versus 13.95% and 9.30%). Reasonable explanations are: (1) Firstly, the anti-rK39 IgG antibodies were only detected by rK39 dip-stick when active infection occurs [20]. Thus, the sensitivity of rK39 dip-stick would be restricted when applied in non-active infection stages; (2) Secondly, the ELISA method detects *Leishmania* antigens via specific antigen-antibody reaction. However, if the load of *Leishmania* in samples is too low to be detected, the usefulness of the ELISA method will be limited; (3) Most importantly, a recombinant antigen of *L. chagasi* was used in rK39 dip-stick, while an anti-*L. donovani* antibody was used in the ELISA method. As both *L. chagasi* and *L. donovani* belong to *L. donovani* complex, it would be unreliable to use these methods to detect leishmaniasis caused by other *Leishmania* species [44]. In comparison to the studies of Wang and Shang [20,43], our PCR test result showed lower positive rates (22.03% versus 51.88% and 24.8%). A reasonable deduction is that we chose newly described 7SL RNA as PCR target rather than traditional, sensitive kDNA minicycle. However, 7SL RNA has an advantage over kDNA minicycle, as it can clearly differentiate *Leishmania* species, while the genus-specific kDNA minicycle is relatively inefficient in species discrimination [45,46]. It is quite useful in our study, as our knowledge of *Leishmania* strain prevalent in China is scarce. However, the sensitivity of conventional PCR methods are usually low, new and advanced methods should be used, such as High-resolution melt analysis PCR (HRM PCR) [47], Real-Time PCR [48], reverse dot blot assay [29] which can vastly enhance sensitivity. In total, 37.21% of the dogs tested were positive for one or more tests, which highlight the potential risk of leishmaniasis outbreak in this area. Further surveillances and control measures are urgently needed.

The most reliable marker for the species discrimination and phylogenetic analysis within the genus *Leishmania* is *Hsp70* gene [49], and phylogenetic tree based on 7SL RNA [31] shared a similar topology with the tree based on *Hsp70*, which ensures the phylogenetic analysis based on 7SL RNA is robust. In our analysis, the phylogenic tree based on 7SL RNA also showed a similar topology to the tree based on *Hsp70*[49]. Interestingly, the 7SL RNA sequences obtained in this study did not form a clade with any known pathogenic *Leishmania* species, but formed a clade, which was most closely related to the nonpathogenic *L. tarentolae*. The strain MHOM/CN/90/SC10H2, which was isolated in a VL patient, was also in this clade. The topology is congruent with our previous studies based on COII, 18 S rRNA and 7SL RNA [17,19]. This result provides evidence that an undescribed *Leishmania* species, which is clearly a causative agent of CanL and human VL, does exist in China. Although the phylogeny based on 7SL RNA indicates these unisolated *Leishmania* are most closely related to *L*. *tarentolae*, however, our knowledge on the geographical distribution of *L. tarentolae* is rare and valid *L. tarentolae* strains are scarce. In the meantime, we are also unclear of the geographical distribution of the *Leishmania* sp*.*, as known *Leishmania* sp*.* strains were rare. Therefore, as the genetic relationship is more related to geography rather than clinical effects [50], it would be hasty to deduce the evolutionary relationship between these two. Thus, more isolates of *Leishmania* sp*.* and *L. tarentolae* from different areas would add to our understanding of this issue. Also, traditional multilocus enzyme electrophoresis (MLEE) methods and newly designed Multilocus Sequence Analysis (MLSA) methods must be combined to validate this undescribed *Leishmania* sp. in China.

## Conclusions

In conclusion, we investigated the prevalence of canine leishmaniasis in Beichuan County, Sichuan Province, China, based on rK39 dip-stick, ELISA and PCR methods. A total of 86 dogs were tested. Among them, 13 dogs were positive using the Dipstick test (15.12%), while 8 dogs were positive using the ELISA test (9.3%) and 19 dogs were positive for PCR (22.03%). Global prevalence, 32/86 (37.21%) dogs were positive for one or more tests. Interestingly, phylogeny based on the partial 7SL RNA segments showed these unisolated *Leishmania* did not belong to any known pathogenic *Leishmania* species, but formed a distinct group closely related to nonpathogenic *L. tarentolae*. Our findings provide evidence that: (1) an undescribed *Leishmania* species, which is clearly a causative agent of CanL and human VL, does exist in China; (2) the prevalence of canine leishmaniasis is very high in Beichuan County, further surveillance and control measures are urgently needed.

## Competing interests

The authors declare that they have no competing interests.

## Authors’ contributions

KS and WG carried out the study, participated in the sequence alignment, phylogenetic analysis and drafted the manuscript. LL carried out the culture of *Leishmania* strain MHOM/CN/90/SC10H2. JGZ and BBY participated in the ELISA assay. YT and YJW participated in the sequence alignment and phylogenetic analysis. DLC and JPC designed the study and participated in drafting the manuscript. All authors read and approved the final version of this manuscript.
